# QuickStats

**Published:** 2015-03-27

**Authors:** FROM THE NATIONAL CENTER FOR HEALTH STATISTICS

**Figure:**
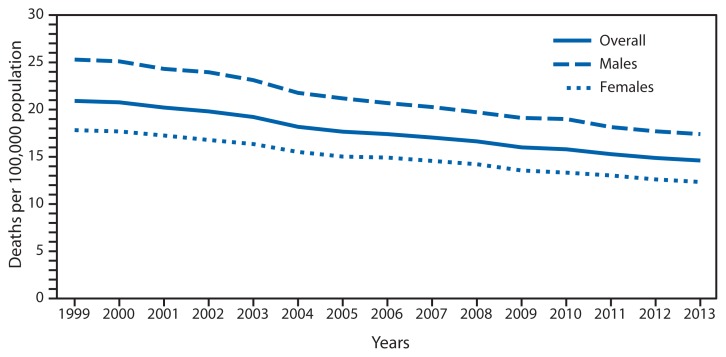
Colorectal Cancer* Death Rates,^†^ by Sex — National Vital Statistics System, United States, 1999–2013 * Malignant neoplasms of colon, rectum and anus (C18–C21) as the underlying cause of death includes the following *International Classification of Diseases, 10th Revision* codes: malignant neoplasm of colon (C18), malignant neoplasm of rectosigmoid junction (C19), malignant neoplasm of rectum (C20), and malignant neoplasm of anus and anal canal (C21). ^†^ Age-adjusted rates (deaths per 100,000) based on the 2000 U.S. standard population. Populations used for computing death rates for 2011–2013 are postcensal estimates based on the 2010 census, estimated as of July 1, 2013. Rates for census years are based on populations enumerated in the corresponding censuses. Rates for noncensus years before 2010 are revised using updated intercensal population estimates and might differ from rates previously published.

In 2013, the age-adjusted death rate for colorectal cancer was 14.6 per 100,000 population, the lowest rate ever recorded. From 1999 to 2013, colorectal cancer death rates decreased 30.1% (from 20.9 to 14.6 per 100,000 population). For males, the rate decreased 31.2%, and for females the rate decreased 30.9%. In 2013, a total of 52,252 colorectal cancer deaths were reported in the United States.

**Source:** National Vital Statistics System. Mortality public use data files, 2013. Available at http://www.cdc.gov/nchs/data_access/vitalstatsonline.htm.

**Reported by:** Betzaida Tejada-Vera, MS, fsz2@cdc.gov, 301-458-4231.

